# A Case Report of Extrapelvic Endometriosis: Surgeon's Perspective and Review of the Literature

**DOI:** 10.7759/cureus.88319

**Published:** 2025-07-19

**Authors:** Prabu Shankar Sivagnanam, Husham Bakry, Aysha Aljawder

**Affiliations:** 1 General Surgery, King Hamad University Hospital, Bahrain Defence Force Royal Medical Services, Al Sayh, BHR; 2 Pathology, King Hamad University Hospital, Bahrain Defence Force Royal Medical Services, Al Sayh, BHR

**Keywords:** abdominal wall, appendiceal, cesarean section scar, endometriosis, extrapelvic

## Abstract

Endometriosis, while commonly managed by gynecologists, is rarely encountered by general surgeons and is often identified incidentally or upon histopathological examination. This condition can manifest in both pelvic and extrapelvic sites, with extrapelvic endometriosis most frequently observed in the gastrointestinal tract and abdominal wall. Cesarean scar endometriosis (CSE) is the most prevalent form of abdominal wall endometriosis. Cyclical symptoms linked to menstruation occur in around half of patients and should raise suspicion in cases presenting with scar-related or subcutaneous swellings. This report presents two cases of extrapelvic endometriosis: one involving appendiceal endometriosis, which was unexpectedly identified as appendicitis, and another with CSE, diagnosed after surgical excision of a painful swelling in the cesarean section scar. The discussion focuses on the diagnosis and management of CSE and appendiceal endometriosis.

## Introduction

Endometriosis is a chronic, estrogen-sensitive condition wherein endometrial glands and stroma are found outside the uterine cavity. Although predominantly observed in pelvic locations such as the ovaries, uterine ligaments, and pelvic peritoneum, ectopic endometrial implants can also occur in distant regions, collectively known as extrapelvic endometriosis. This atypical variant encompasses lesions within the gastrointestinal (GI) and urinary tracts, thoracic cavity, abdominal wall, and even the nervous system. A classification scheme proposed by Markham et al. segments extrapelvic endometriosis into four categories: gastrointestinal, urinary, thoracic, and "other" sites [1].

Among extrapelvic locations, the GI tract is most commonly involved, likely due to its anatomical proximity to the uterus and fallopian tubes [2]. Within the GI system, the appendix is an uncommon but recognized site, with an estimated prevalence of approximately 2.7% among women undergoing appendectomy for right lower-quadrant pain [3]. Despite this rarity, appendiceal endometriosis can present clinically with symptoms that mimic acute appendicitis, frequently identified only after histological examination following removal.

In the abdominal wall, scar-related endometriosis, especially at the cesarean section incision, is the most frequently reported form of extrapelvic disease. It typically presents years after surgery as a palpable mass and cyclical pain, and occurs in 0.03-3.5% of post-cesarean patients [4,5].

## Case presentation

Case 1: appendiceal endometriosis

A 43-year-old woman with no significant medical history presented to the emergency department with three days of generalized lower abdominal pain (colicky, intermittent), nausea, anorexia, one episode of loose stool, and subjective low-grade fever. She had no urinary symptoms or any gynecological symptoms like dysmenorrhea or vaginal discharge. She had regular menstrual cycles with normal flow. Her last menstrual cycle was one week prior to presentation. She had three children delivered by normal vaginal delivery with no past history of miscarriages. On physical examination, she had tenderness in the right iliac fossa with rebound tenderness, and no palpable mass or signs of peritonitis.

Laboratory tests (Table 1) revealed elevated inflammatory markers, which suggested an inflammatory process consistent with acute appendicitis.

**Table 1:** Laboratory tests.

Blood test (units)	Result	Reference range
Total leucocyte count (x 10^9/L)	14.42	4-11
Neutrophil percentage (%)	83.5	-
Absolute neutrophil count (x 10^9/L)	11.1	1.5-8
C-reactive protein (mg/L)	66.2	0.0-10

Subsequent imaging included an abdominal and pelvic ultrasound in which the appendix could not be visualized, and there was no evidence of ovarian cyst or torsion, but noted the presence of multiple small uterine fibroids.

**Figure 1:**
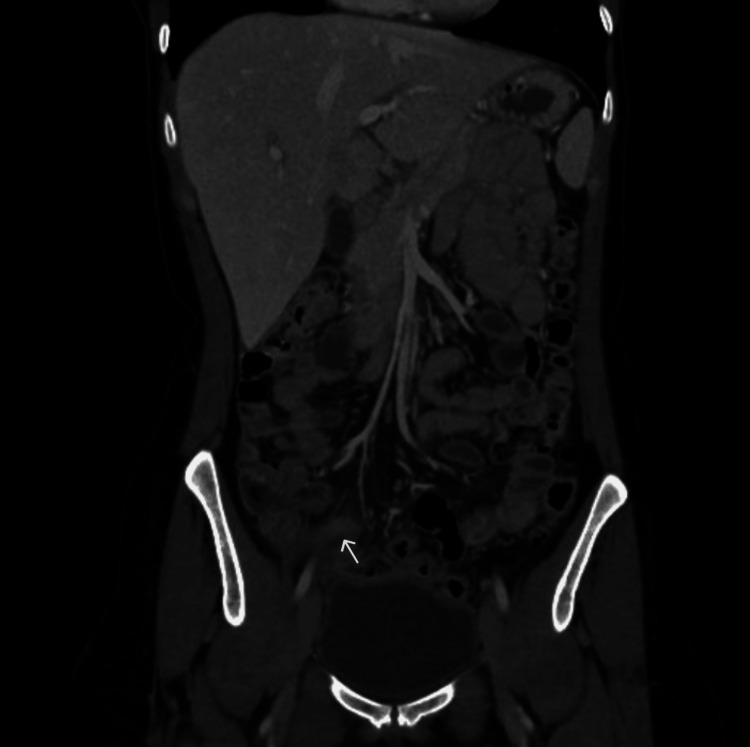
Contrast-enhanced CT of the abdomen and pelvis showing thickened and enlarged appendix (white arrow).

A contrast-enhanced CT scan of the abdomen and pelvis (Figure 1) was performed, revealing an enlarged and thickened vermiform appendix measuring approximately 8 mm in diameter, with a fluid-filled tip and haziness in the surrounding fat, suggestive of acute appendicitis. Additionally, several enlarged mesenteric lymph nodes were identified, with the largest measuring 11.5 x 7 mm.

**Figure 2:**
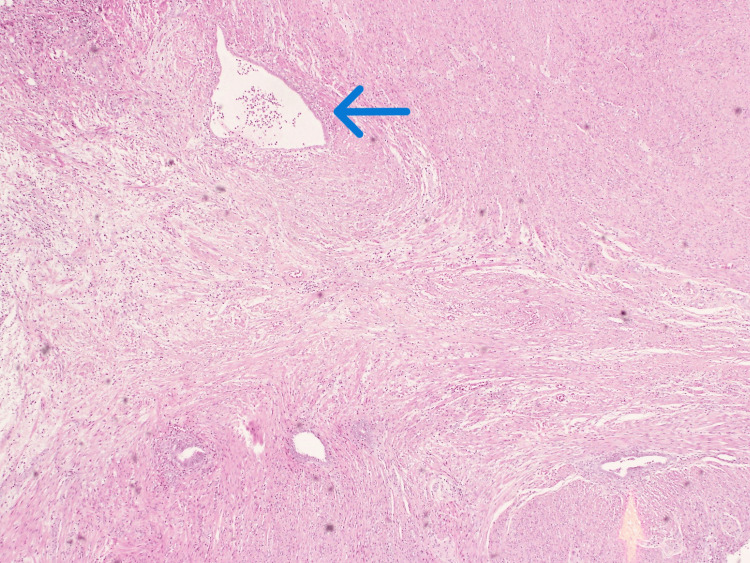
Hematoxylin & eosin (H&E) staining showing scattered islands of endometrial glands rimmed by stroma (blue arrow) within the appendiceal wall.

**Figure 3:**
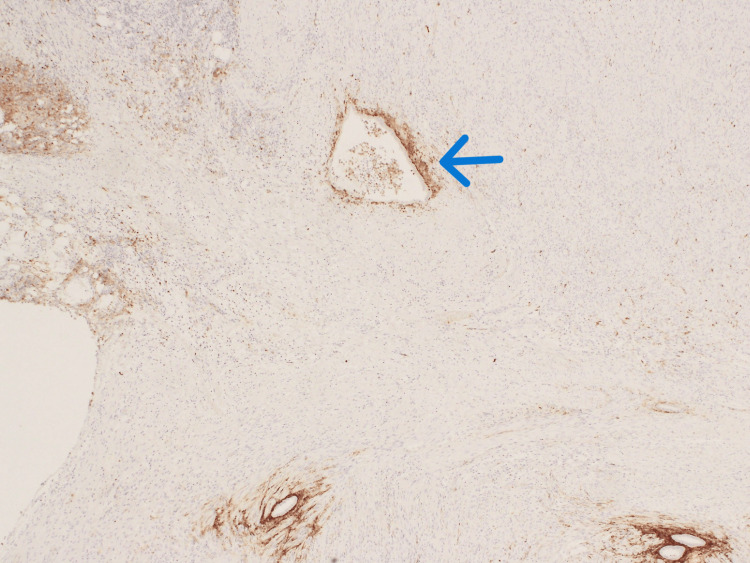
CD10 immunohistochemistry staining showing the endometrial stromal component (blue arrow) of the scattered glandular islands.

**Figure 4:**
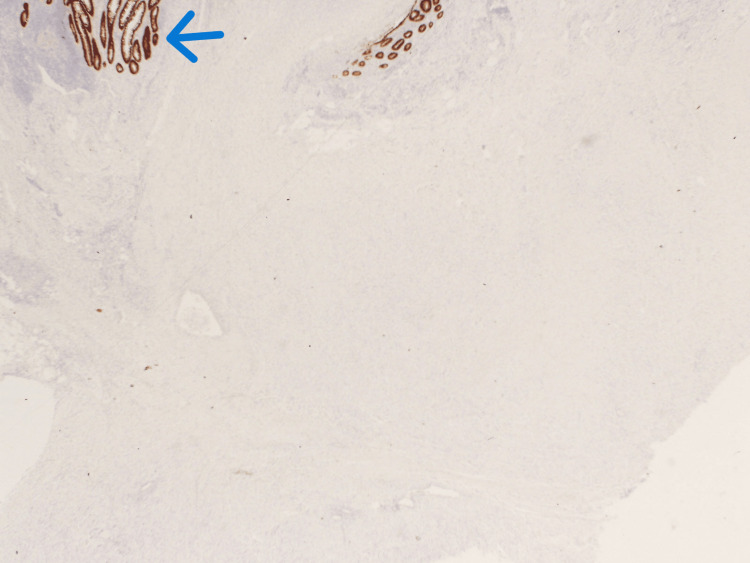
CDX2 immunohistochemistry staining of the colorectal mucosa showing only the appendiceal normal mucosal elements (blue arrow) working as an internal control.

Given the clinical picture of acute appendicitis, the patient underwent a laparoscopic appendectomy. Intra-operatively, the appendix was found to be inflamed and situated in a pelvic position, with pus flakes covering its surface and adherent to the right fallopian tube and retroperitoneum. Turbid fluid was found in the pouch of Douglas, but the terminal ileum and cecum were normal. The posterior wall of the uterus showed a few small fibroids, while the ovaries were assessed to be normal with no cystic changes. Adhesiolysis was performed. The appendectomy was performed successfully without complications, and the patient tolerated the operation well. She was discharged the following day with no postoperative complications. Histopathological examination (Figures 2-4) of the resected appendix revealed intact colorectal-type mucosa with scattered endometrial-type glands and minimal endometrial stroma, confirming the diagnosis of appendiceal endometriosis. There was no histological evidence of acute inflammation. Immunohistochemical staining confirmed the presence of endometrial tissue without any dysplasia or malignancy.

The patient was referred to a gynecologist for further management.

Case 2: cesarean scar endometriosis (CSE)

A 33-year-old female, with a past surgical history of cesarean section done four years prior to presentation, presented with a one-year history of swelling along the left side of her C-section scar. The swelling was described as non-progressive, non-reducible, and was associated with mild pain that intensified during menstruation. The patient reported regular menstrual cycles without any accompanying symptoms such as loss of appetite, weight loss, or changes in bowel or bladder habits.

On examination, the abdomen was soft and lax, with a 1 x 1 cm swelling along the left side of the C-section scar. The swelling was firm, fixed to the rectus sheath, and exhibited no overlying skin changes or signs of inflammation. There was no expansile cough impulse. She was further worked up with a differential diagnosis of scar endometriosis, suture granuloma, or soft tissue tumor.

**Figure 5:**
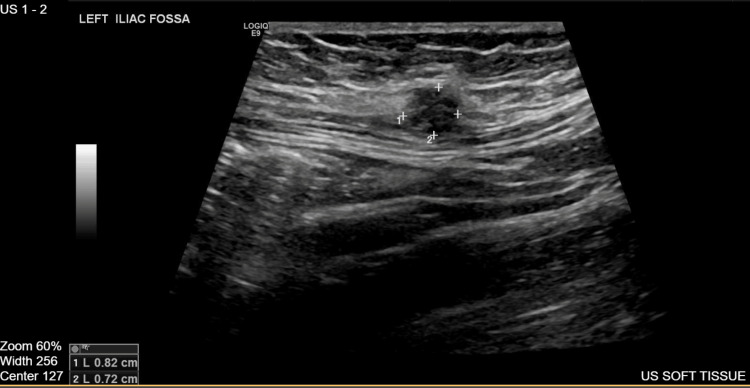
Soft tissue ultrasound showing a cystic lesion in the left rectus muscle.

She underwent a soft tissue ultrasound (Figure 5) of the abdominal wall, which identified a well-defined cystic lesion measuring 8 x 7 mm with echogenic contents, located overlying the left rectus abdominis muscle adjacent to the cesarean section scar. The findings were suggestive of scar endometriosis and required further clinical correlation.

**Figure 6:**
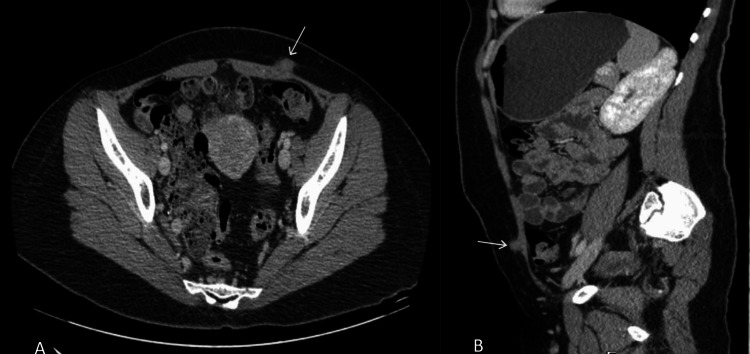
Contrast-enhanced CT of the abdomen showing a soft tissue mass in the left rectus abdominis muscle in axial (A) and sagittal (B) sections.

Contrast-enhanced CT scan of the abdomen (Figure 6) revealed a small soft tissue mass arising from the left rectus abdominis muscle, measuring 15 x 12 mm. There was no evidence of deeper extension into the abdominal cavity, and no suture material was detected as a dense foreign body. Additionally, a large left adnexal cyst was identified, measuring 45 x 42 x 35 mm, prompting further correlation via ultrasound.

The patient subsequently became pregnant and remained asymptomatic with no change in size of the swelling during her antenatal period, ultimately having a normal vaginal delivery and lost follow-up. However, after two years post delivery, she returned for follow-up, reporting symptoms of increasing pain during her menstrual cycles and a static size of the abdominal wall swelling.

**Figure 7:**
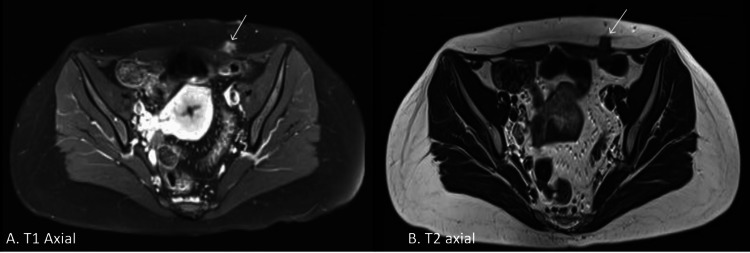
MRI of the soft tissue of the abdominal wall showing a soft tissue lesion in T1 axial (A) and T2 axial (B) sections.

MRI of the soft tissue (Figure 7) revealed a small, well-defined, enhanced soft tissue lesion in the lower anterior abdominal wall on the left side. The lesion was located in the deep subcutaneous plane, based on the lateral aspect of the left rectus abdominis muscle, measuring 14 x 13 x 11 mm. The imaging demonstrated iso- to hypointense signals in T1 and T2-weighted images, with a few peripheral fine linear enhancing reticulations and thin linear enhancement along the deep fascial plane. Importantly, there was no intra-abdominal extension. The MRI features suggested a small desmoid tumor, necessitating histopathological correlation.

The patient underwent a wide local excision of the soft tissue swelling in the left abdominal wall under general anesthesia. Intraoperative findings indicated a 4 x 3 cm swelling with firm consistency and diffuse borders, adherent to the anterior rectus fascia in the left lower abdominal wall, with primary repair of the anterior rectus sheath with delayed absorbable suture material.

**Figure 8:**
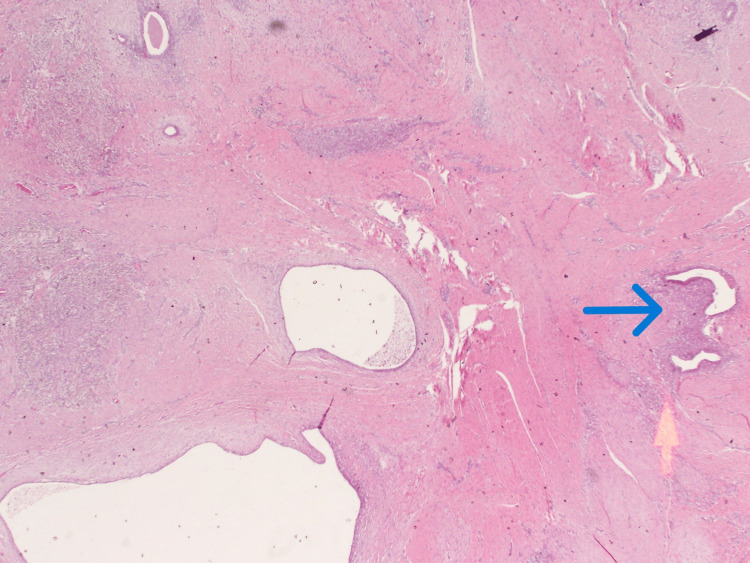
Hematoxylin & eosin (H&E) staining showing islands of endometrial glands and stroma (blue arrow).

**Figure 9:**
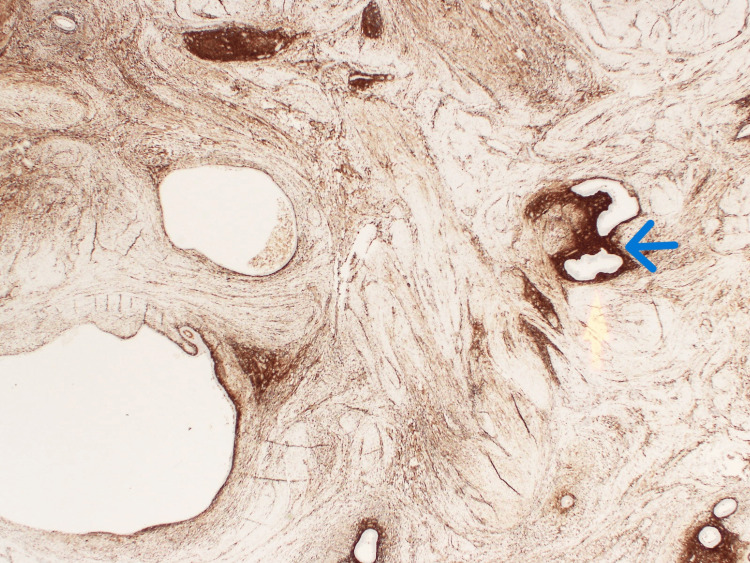
CD10 immunohistochemistry staining highlighting the endometrial stromal elements (blue arrow).

**Figure 10:**
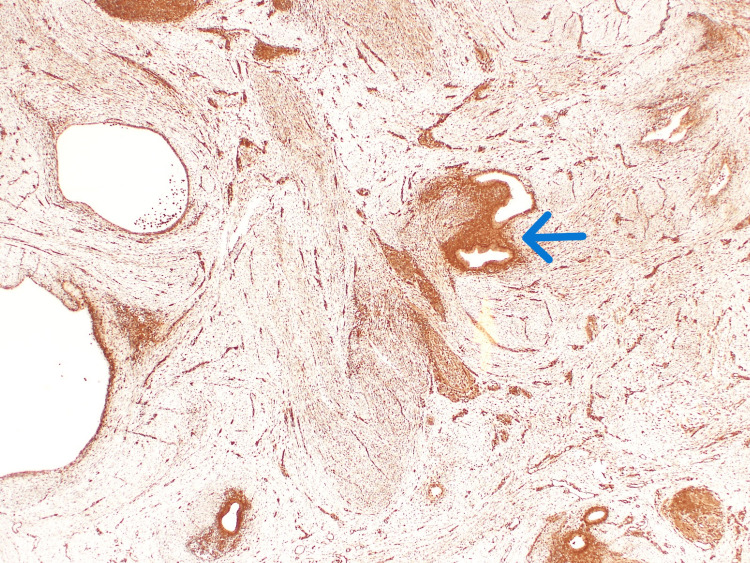
Vimentin immunohistochemistry staining confirming the endometrial glands and stroma (blue arrow).

The histopathological examination of the excised tissue (Figures 8-10) showed a firm fibrotic tan lesion measuring 14 x 12 x 11 mm, with microscopic interpretation showing fibromuscular tissue containing endometrial glands, endometrial stroma, and areas of hemorrhage. Some glands exhibited intraluminal macrophages, but there was no evidence of atypia or malignancy, confirming the diagnosis of scar endometriosis.

The patient was referred to a gynecologist for further management, and the patient underwent an ultrasound of the pelvis, which showed no ovarian pathology.

These cases highlight two uncommon presentations of extra-pelvic endometriosis, emphasizing the need for histopathological confirmation for accurate diagnosis and the importance of distinguishing between types of endometriosis for targeted management.

## Discussion

In these cases, we highlighted two rare presentations of extrapelvic endometriosis: appendiceal endometriosis and CSE. Extrapelvic endometriosis is an uncommon variant of endometriosis, a condition that generally affects the pelvic region, particularly in areas such as the ovaries, fallopian tubes, and peritoneum. In these cases, endometriotic implants occurred outside of the pelvis, affecting the appendix and abdominal wall.

Case analysis

Case 1 demonstrates appendiceal endometriosis presenting as acute appendicitis. Appendiceal endometriosis is rare, with an estimated prevalence of around 2.8% among patients with endometriosis [6]. The gastrointestinal tract has been the most common site of extrapelvic endometriosis because of its proximity to the fallopian tubes. The rectum, sigmoid, and terminal ileum are the common sites in intestinal endometriosis, with appendiceal endometriosis contributing by 5-20% [2]. Two main hypotheses have been proposed to explain its cause: one suggests that multipotential mesenchymal cells may undergo metaplasia into endometriosis under proper circumstances; the other hypothesis states that viable endometrial cells are implanted from retrograde menstruation through the fallopian tubes [6]. Symptoms can mimic acute or chronic appendicitis or can be entirely asymptomatic, as often seen when the condition is diagnosed incidentally during histopathological examinations. In this case, the patient presented with classic appendicitis symptoms, and a laparoscopic appendectomy was performed. Histopathological examination revealed endometrial glands and stroma within the appendix, confirming appendiceal endometriosis. Although imaging techniques like CT scans and ultrasound can assist in assessing inflammation, no imaging modality is definitive for diagnosing endometriosis. Histopathology remains the gold standard, confirming the diagnosis and guiding the need for further management.

Case 2 presents a case of CSE, a type of abdominal wall endometriosis that is typically associated with prior surgical interventions, such as a cesarean section. Scar endometriosis likely occurs due to the inadvertent implantation of endometrial tissue into the wound during surgery, which then becomes hormonally responsive, causing cyclical pain during menstruation [7]. In this case, the patient presented with a painful, cyclical swelling near her cesarean scar that was diagnosed based on clinical suspicion, imaging, and ultimately confirmed through histopathological examination. The patient underwent a wide local excision with clear margins, which remains the treatment of choice to prevent recurrence.

Diagnostic considerations

The diagnosis of extrapelvic endometriosis is often challenging, especially given that symptoms can mimic other conditions. In cases like appendiceal endometriosis, the clinical presentation may align with acute appendicitis, but imaging cannot definitively diagnose the condition. Instead, histopathological examination after surgery confirmed the presence of ectopic endometrial tissue. A preoperative diagnosis is difficult but should be included in the differential diagnosis when women of childbearing age present with clinical symptoms of acute appendicitis or present with nonspecific recurrent lower abdominal pain, especially with a history of menstrual irregularities or infertility [8]. It is very important to diagnose endometriosis of the appendix early so that patients can receive the treatment they need. If left untreated, appendiceal endometriosis can lead to bleeding or perforation in the intestines and obstruction of the bowels.

Similarly, CSE is often misdiagnosed as a suture granuloma, incisional hernia, or other soft tissue lesion. Clinical suspicion, along with a history of previous pelvic or abdominal surgery and cyclical symptoms related to menstruation, should raise the possibility of scar endometriosis [5].

Management strategies

Surgical resection remains the definitive treatment for both appendiceal endometriosis and abdominal wall endometriosis, as in our cases. For appendiceal endometriosis, an appendectomy is usually sufficient in cases without extensive disease. If other endometriotic implants are detected, a more extensive surgical intervention or medical therapy may be necessary. For CSE, wide local excision with adequate margins is recommended to minimize the risk of recurrence [9]. Hormonal therapy is generally not preferred as it may only offer partial symptom relief without addressing the root cause, and recurrence is likely once treatment is stopped.

Preventive measures for scar endometriosis

Scar endometriosis can be reduced by implementing preventive surgical techniques. During procedures such as cesarean sections or gynecological surgeries, thoroughly irrigating the surgical site and changing gloves before wound closure can reduce the risk of endometrial cell implantation [10]. Additionally, repairing the peritoneum has been suggested to prevent abdominal wall implantation [11].

Implications

These cases underline the diversity of endometriosis presentations and emphasize the need for awareness among surgeons and gynecologists. Further research on the pathogenesis and epidemiology of extrapelvic endometriosis is necessary to improve early diagnosis and optimize management strategies for patients presenting with atypical symptoms and to better understand these rare cases. It would be advisable to develop a registry through multidisciplinary collaboration involving gynecologists, surgeons, and clinicians managing these patients [12].

## Conclusions

Extrapelvic endometriosis, although rare, should be considered in differential diagnoses, especially in patients with a history of prior pelvic or abdominal surgery. While endometriosis is commonly seen in women of reproductive age, its extrapelvic manifestations are uncommon and often overlooked by general surgeons. A meticulous patient history, with specific attention to previous surgical procedures and cyclical symptomatology, is crucial in raising clinical suspicion and guiding appropriate workup. Surgical resection remains the mainstay of treatment, especially for cases such as cesarean scar endometriosis and appendiceal involvement. Implementing preventive surgical techniques such as thorough irrigation of the operative field and careful closure techniques can help reduce the risk of iatrogenic endometrial implantation. With increased clinical awareness and precise diagnostic approaches, healthcare providers can significantly improve outcomes for patients presenting with these atypical and often challenging forms of endometriosis.
